# Deciphering Breast Cancer Complexity: A Study on the Predictive Power of MRI Texture Analysis for Tumor Characterization and Treatment Response

**DOI:** 10.5334/jbsr.3913

**Published:** 2025-12-23

**Authors:** Hamza Eren Güzel, Ali Murat Koç, Zehra Hilal Adıbelli, Funda Taşlı, Babak Saravi

**Affiliations:** 1 Department of Radiology, İzmir City Hospital, İzmir, Turkey; 2 Department of Radiology, Atatürk Education and Research Hospital, İzmir Katip Celebi University, İzmir, Turkey; 3 Department of Oral, Maxillofacial and Facial Plastic Surgery, Medical Faculty and University Hospital Düsseldorf, Heinrich-Heine-University Düsseldorf, Düsseldorf, Germany; 4 Department of Pathology, İzmir City Hospital, İzmir, Turkey

**Keywords:** breast cancer, MRI, texture, subtype, correlation

## Abstract

*Objectives:* This study aimed to examine the association between MRI radiomics features of malignant breast masses and their histopathological, molecular characteristics, and response to neoadjuvant treatments.

*Materials and methods:* A retrospective cohort of 70 breast cancer patients was analyzed. Texture analysis was performed on preoperative MRI scans, extracting features such as entropy, contrast, and homogeneity. These features were analyzed against histopathological and clinical targets (e.g., lymph node metastasis) and molecular profiles. Statistical analyses and machine learning algorithms, including logistic regression and support vector machines, were employed to evaluate the predictive power of MRI texture features for molecular subtypes and the association of radiomics markers with neoadjuvant treatments.

*Results:* The findings revealed significant associations between MRI texture features and the histopathological and molecular characteristics of breast tumors. Certain texture parameters correlated with aggressive phenotypes and poor chemotherapy response. Despite the limited dataset, machine learning models performed well in classifying tumors and predicting outcomes, highlighting the potential of MRI texture analysis in clinical decision‑making.

*Conclusion:* MRI texture analysis emerges as a non‑invasive tool for personalized breast cancer management. The significant associations between MRI texture features and critical tumor characteristics suggest that these features could serve as biomarkers for predicting tumor behavior and treatment efficacy. Further large‑scale research is needed to integrate MRI texture analysis into clinical practice and improve patient outcomes.

## 1. Introduction

In 2020, female breast cancer became the leading cause of cancer incidence globally, with approximately 2.3 million new cases, accounting for 11.7% of all cancer cases and making it the most commonly diagnosed cancer in women. It is also the leading cause of cancer‑related deaths among them, with incidence rates of 55.9 per 100,000 in developed countries and 29.7 per 100,000 in developing countries [1]. To combat this, global screening programs focus on early detection to reduce mortality [2]. The World Health Organization recommends biennial mammography screenings for women aged 50 to 69 at average risk, particularly in resource‑rich settings [3].

Mammography remains the cornerstone of breast cancer screening, while diagnostic evaluations utilize advanced imaging techniques such as specialized mammography, ultrasonography (USG), and magnetic resonance imaging (MRI). MRI, particularly dynamic contrast‑enhanced MRI, is highly accurate for invasive breast carcinomas, with sensitivity ranging from 75.2% to 100% and specificity between 83% and 98.4% [4]. It goes beyond lesion detection, analyzing morphology, contour, volumetric assessments, diffusion limitations, and contrast uptake patterns, which help differentiate benign from malignant lesions and predict their histopathological features [5].

Radiomics, an emerging field in medical imaging, extracts quantitative data from routine images and offers significant advantages over traditional biopsy methods by enabling non‑invasive analysis of entire tumor phenotypes, unlike biopsies, which only sample small portions of a tumor [6, 7]. Radiomics can be applied to breast cancer diagnostics to predict tumor characteristics, response to therapy, molecular subtypes, and lymph node metastases [8–11]. Traditional diagnostic methods, relying on mammography, ultrasound, and MRI, have limitations such as suboptimal sensitivity, invasive biopsies, and prolonged waiting times for results [12–15]. Radiomics offers a non‑invasive, comprehensive approach that supports personalized treatment strategies and decision‑making, reducing the need for invasive procedures [16].

The primary objective of our study is to explore the correlation between MRI‑derived texture features of malignant breast masses and their histopathological, molecular characteristics, and response to neoadjuvant treatments. Our goal is to assess the utility of these MRI features as indicators of intratumoral heterogeneity, ultimately advancing personalized breast cancer management.

## 2. Materials and Methods

### 2.1 Study design and population

The research focused on breast cancer patients diagnosed in the Department of Pathology at the same institution in 2018 and 2019. Inclusion criteria were: (1) histopathologically confirmed breast carcinoma; (2) surgical intervention for breast cancer at our facility; (3) preoperative breast MRI scans at our institution; and (4) lesions ≥1 cm suitable for texture analysis. A total of 70 eligible cases were included.

Morphological and molecular data from pathology reports were extracted, and a comprehensive texture analysis of the MRI scans was conducted to explore potential correlations between radiomic features and pathological findings.

### 2.2 Histopathological findings and study variables

Histopathological analyses were performed on specimens from partial or total mastectomies conducted between October 7, 2020, and January 10, 2021. Tissue samples were fixed in 10% formalin, embedded in paraffin, and sectioned into 4‑micron slices for hematoxylin and eosin staining. These were examined for histological grade and lymphovascular invasion.

Immunohistochemical markers (ER, PR, Cerb B2, p53, Ki‑67) were evaluated using an automated staining system (Ventana BenchMark XT). Pathological data included: (1) receptor characteristics, categorized by intrinsic subtypes (Luminal A, Luminal B, HER2 overexpression, and triple‑negative); (2) Ki‑67 index classification (low <30%, high ≥30%); (3) Bloom–Richardson grading; (4) p53 positivity (negative <5%, weakly positive 5–50%, strongly positive >50%); (5) lymphovascular invasion; (6) lymph node metastasis; and (7) neoadjuvant treatment response (Miller–Payne system) for 20 patients, categorized as ‘no response’ (grades 1–3) or ‘response’ (grades 4–5) (Table 1).

**Table 1:** Miller‑Payne scoring system for evaluating pathological response to neoadjuvant treatment [17].

GRADE	DESCRIPTION
1	Minimal or no cellular‑level changes, with unchanged overall cell density
2	Up to 30% reduction in tumor cell density
3	Reduction in tumor cell density ranging from 30 to 90%
4	Greater than 90% decrease in tumor cell density, with cells discernible individually or in small clusters
5	Complete absence of malignant cells within the tumor bed

### 2.3 MRI procedure

Breast MRI exams were conducted using a 1.5 Tesla MRI system (Magnetom AERA, Siemens, Germany). An 8‑channel surface breast coil was used, with imaging covering a 32‑cm field. An antecubital vein was cannulated for gadolinium‑based contrast (meglumine gadoterate—Dotarem) at a dose of 0.1–0.2 mmol/kg.

The protocol included localizer images, followed by axial fat‑suppressed turbo spin echo (TSE) T1‑weighted, turbo inversion recovery magnitude (TIRM), and T2‑weighted sequences. Dynamic imaging was performed with post‑contrast T1‑weighted images using SPAIR sequences in axial and sagittal planes, with six repetitions at 1‑min intervals.

Key imaging parameters were: TSE T1A (TR 476 ms, TE 11 ms, matrix 384 × 297, NEX 1, 4 mm slice thickness), TIRM T2A (TR 2250 ms, TE 56 ms, TI 165 ms, matrix 384 × 270, NEX 1, 4 mm slice thickness), axial T2A TSE (TR 5350 ms, TE 76 ms, matrix 320 × 217, NEX 2, 4 mm slice thickness), and dynamic T1A (TR 4.53 ms, TE 1.82 ms, flip angle 10°, matrix 416 × 313, NEX 1, 2 mm slice thickness). Diffusion‑weighted imaging (DWI) used the echo‑planar technique with *b*‑values of 50, 200, and 800 s/mm², with TR 6400 ms, TE 66 ms, matrix 220 × 84, NEX 2, 4 mm slice thickness.

### 2.4 Radiologist evaluation and lesion characterization

Two radiologists evaluated all MR images. The assessed features included:

1. Breast composition: Classified according to a four‑point scale of BI‑RADS A to D.
2. Background contrast enhancement: Non‑pathological enhancement of fibroglandular tissue, categorized into four levels: minimal (<25%), light (25–50%), medium (50–75%), and distinctive (>75%).
3. Lesion characteristics:
  - Location: Upper outer quadrant, upper inner quadrant, retroareolar, lower outer quadrant, lower inner quadrant.
  - Volume: Determined by volume measurement and expressed in mm³.
  - Shape: Round, oval, irregular.
  - Edge: Sharp, veiled, microlobulated, indistinct, spiculated.
  - T1 Signal: Isointense, hypointense, hyperintense relative to parenchyma.
  - T2 Signal: Isointense, hypointense, hyperintense relative to parenchyma.
  - Contrast enhancement pattern: Homogeneous, contrast enhancing septa, heterogeneous, none, peripheral.
  - Diffusion property: Compared to parenchyma (less, equal, more).
  - Contrast enhancement curve: Type 1, 2, or 3.
  - ADC value: Recorded.
4. Other lesions: Presence of multifocality and multicentricity.

### 2.5 Radiomics‑Based texture analysis

**Figure 1:**
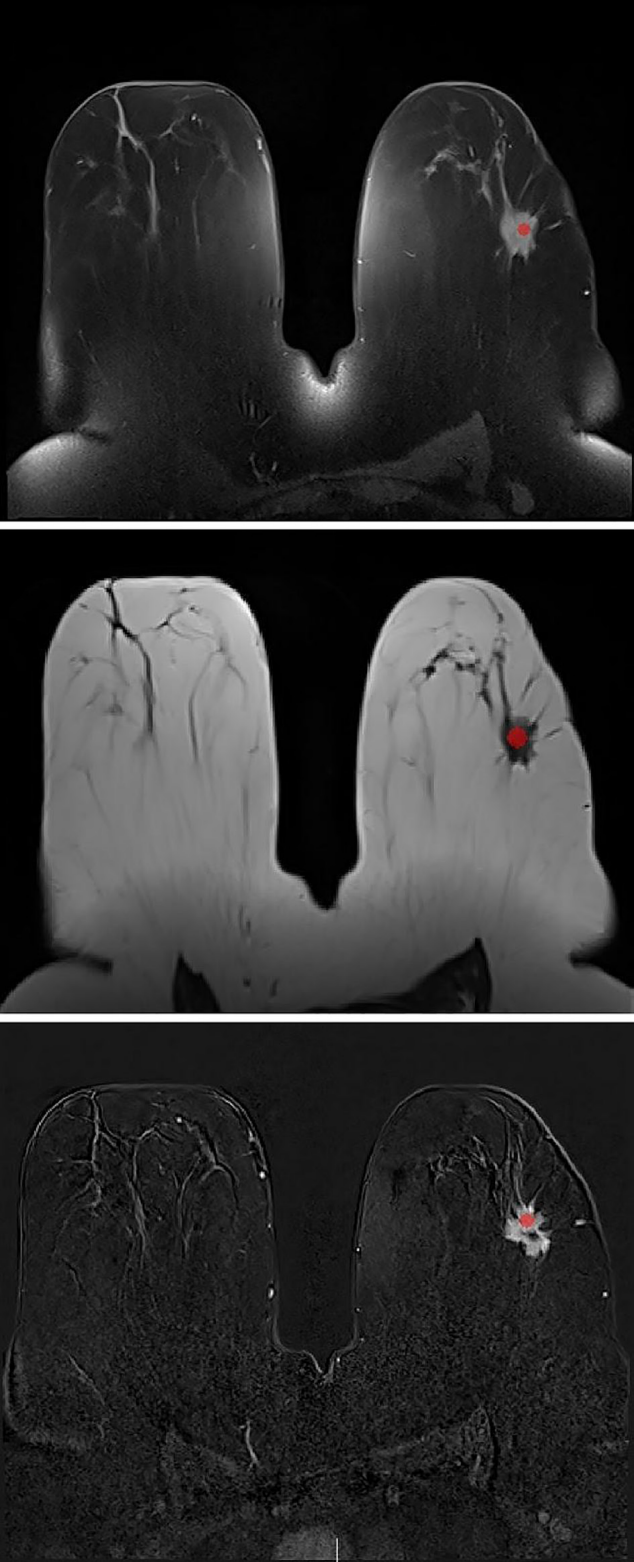
Region‑of‑interest (ROI) placement for texture analysis on T1A, T2A, and postcontrast T1A subtraction images of the same patient.

MRI data of the enrolled cases, encompassing sequences, including T1A, T2A, early and late‑phase post‑contrast T1A, and diffusion‑weighted imaging, were acquired in Digital Imaging and Communications in Medicine (DICOM) format. These images were then processed using the MaZda 4.6 software, a specialized tool for texture analysis developed by Strzelecki et al. [18]. A critical step involved the demarcation of the mass on the images by defining a region of interest (ROI). Recognizing the impact of ROI size on texture analysis outcomes, as evidenced in previous studies [19], a uniform ROI of 10 × 10 pixels was employed for each lesion. Adhering to established protocols, the ROI was strategically placed on the most solid and contrast‑enhanced segment of the lesion (Figure 1) [20].

The MaZda software facilitated a multifaceted texture analysis, starting with a histogram analysis that provides a global assessment based on pixel intensity, independent of spatial pixel relationships. The gradient analysis was executed by computing the gradient histogram of image intensity distribution. Co‑occurrence matrix (COM) analysis was employed to evaluate spatial relations and densities of pixel pairs at varied angles. Additionally, run‑length matrix (RLM) analysis was conducted to assess pixel runs of specific grayscale levels and lengths across four orientations (horizontal, vertical, 45°, and 135°). This comprehensive analysis by the software yielded a detailed report for each case, summarizing diverse texture features. Considering the anticipated homogeneity across four‑directional analyses, these values were consolidated into a singular parameter [21]. Hereafter, three‑dimensional texture analysis was executed on the MR images in DICOM format using the MaZda 4.6 software. This analysis yielded several histogram‑based features, including mean brightness, variance, skewness, and kurtosis. Additionally, 11 features were extracted from the gray‑level COM as part of the second‑order texture analysis. These features included angular second moment (AngScMom), contrast, correlation (Correlat), sum of squares (SumofSqs), inverse difference moment (InvDfMom), sum average (SumAverg), sum variance (SumVarnc), sum entropy (SumEntrp), entropy, difference variance (DifVarnc), and difference entropy (DifEntrp).

### 2.6 Machine learning

We evaluated the predictive power of machine learning models for classifying molecular characteristics (Luminal A, Luminal B, HER2, triple‑negative), p53 expression, lymphovascular invasion, and lymph node metastasis using a dataset of clinical, anatomical, and radiomics features. The dataset included clinical data and radiomics markers from T1‑weighted, T2‑weighted, post‑contrast T1, and DWI sequences.

Data preprocessing included one‑hot encoding for categorical variables and standardization of numerical and radiomics features. A stratified k‑fold cross‑validation (fivefold) was used to assess model performance. Four machine learning models—Logistic Regression, Random Forest, Gradient Boosting, and XGBoost—were evaluated for accuracy, precision, recall, F1 score, and AUC‑ROC.

Feature importance analysis with the Random Forest classifier identified the most influential features. The top ten features were visualized to show their importance. All analyses were conducted using Python.

### 2.7 Statistical analyses

Statistical analyses were performed using the SPSS 22 demo package program. Descriptive analyses in the study presented numerical variables as mean, median, standard deviation, minimum–maximum values, and categorical variables as counts, ratios, and percentages. The normal distribution of the data was tested using the Shapiro–Wilk test. For intergroup comparisons, appropriate analytical tests, such as Kruskal–Wallis (including Dunn’s post hoc test), independent *t*‑test, one‑way ANOVA (including Tukey HSD test), and Mann–Whitney U tests (Bonferroni adjusted), were utilized according to the nature of the variables. In the tests, *p*‑values less than 0.05 were considered statistically significant.

## 3. Results

### 3.1 Descriptive statistics

The demographic, anatomical, and molecular characteristics of the cohort are summarized in Table 2.

**Table 2:** Demographic, anatomical, and molecular characteristics of the cohort.

		MEAN	SD	COUNT	%
Age		55.19	13.31		
Lesion volume		1.15	0.21		
Molecular characteristics	Luminal A			13	18.6
	Luminal B			27	38.6
	HER2‑overexpressed			9	12.9
	Triple (−)			21	30.0
Grade	Low			32	45.7
	High			38	54.3
Ki‑67	Ki‑67−			32	45.7
	Ki‑67+			38	54.3
p53	Negative			43	61.4
	Medium positive			12	17.1
	Strong positive			15	21.4
Cerb‑2	Cerb‑B2−			54	77.1
	Cerb‑B2+			16	22.9
ER	ER−			30	42.9
	ER+			40	57.1
PR	PR−			34	48.6
	PR+			36	51.4
Lymphovascular invasion	No			41	58.6
	Yes			29	41.4
Lymph node metastasis	No			34	48.6
	Yes			36	51.4
Neoadjuvant response	No			11	52.4
	Yes			10	47.6
	No neoadjuvant therapy			49	70.0
Breast pattern	A			8	11.4
	B			32	45.7
	C			20	28.6
	D			10	14.3
Background contrast enhancement	1			32	45.7
	2			19	27.1
	3			17	24.3
	4			2	2.9
Lesion location	Upper outer quadrant			40	57.1
	Upper inner quadrant			16	22.9
	Lower outer quadrant			8	11.4
	Lower inner quadrant			3	4.3
	Retro			3	4.3
Shape	Round			9	12.9
	Oval			1	1.4
	Irregular			60	85.7
Edge	Sharp			3	4.3
	Veiled			0	0.0
	Microlobulated			36	51.4
	Indistinct			1	1.4
	Spiculated			30	42.9
Satellite	No			40	57.1
	yes			30	42.9
Multifocal	No			50	71.4
	Yes			20	28.6
Multicentric	No			60	85.7
	Yes			10	14.3
T1 signal	Iso			45	64.3
	Hypo			1	1.4
	Hyper			24	34.3
T2 signal	Iso			36	51.4
	Hypo			12	17.1
	Hyper			22	31.4
Diffusion restriction	Less than parenchyma			0	0.0
	Equal to parenchyma			0	0.0
	More than parenchyma			70	100.0
Contrast pattern	Homogeneous			4	5.7
	Contrast enhancing septa			0	0.0
	Heterogeneous			59	84.3
	No contrast enhancement			0	0.0
	Peripheral contrast enhancement			7	10.0
Contrast enhancement	Slow			5	7.1
	Medium			3	4.3
	Rapid			62	88.6
Contrast enhancement curve	Type 1			4	5.7
	Type 2			50	71.4
	Type 3			16	22.9

This study analyzed 70 cases, with participants aged 28 to 86 years (mean 55.19 ± 13.31 years). Lesion volumes varied widely (429–125,896 mm³, mean 11,504 mm³).

Tumor subtypes included Luminal A (18.6%), Luminal B (38.6%), HER2‑overexpressed (12.9%), and triple‑negative (30.0%), highlighting molecular diversity. Tumor grading was nearly even, with 45.7% low‑grade and 54.3% high‑grade, mirroring Ki‑67 expression levels.

Molecular markers showed p53 negativity in 61.4%, HER2 positivity in 22.9%, ER positivity in 57.1%, and PR positivity in 51.4%. Lymphovascular invasion and lymph node metastasis were found in 41.4% and 51.4% of cases, respectively. Neoadjuvant therapy was administered to 30%, with 47.6% responding positively.

MRI findings revealed type B breast composition in 45.7%. Lesions were mostly in the upper outer quadrant (57.1%), with multifocality (28.6%) and multicentricity (14.3%). All lesions showed diffusion *restriction*, with 84.3% having heterogeneous contrast patterns and 88.6% exhibiting rapid early enhancement.

Lesions were predominantly irregular (85.7%), with microlobulated (51.4%) or spiculated (42.9%) edges. Satellite nodules appeared in 42.9% of cases. On T1‑weighted imaging, 64.3% were isointense, while T2 signals varied (51.4% isointense, 31.4% hyperintense, 17.1% hypointense). The most common enhancement patterns were heterogeneous (84.3%) or peripheral (10%), with rapid enhancement seen in 88.6% of cases.

### 3.2 Pairwise analyses of radiomics features

**Figure 2:**
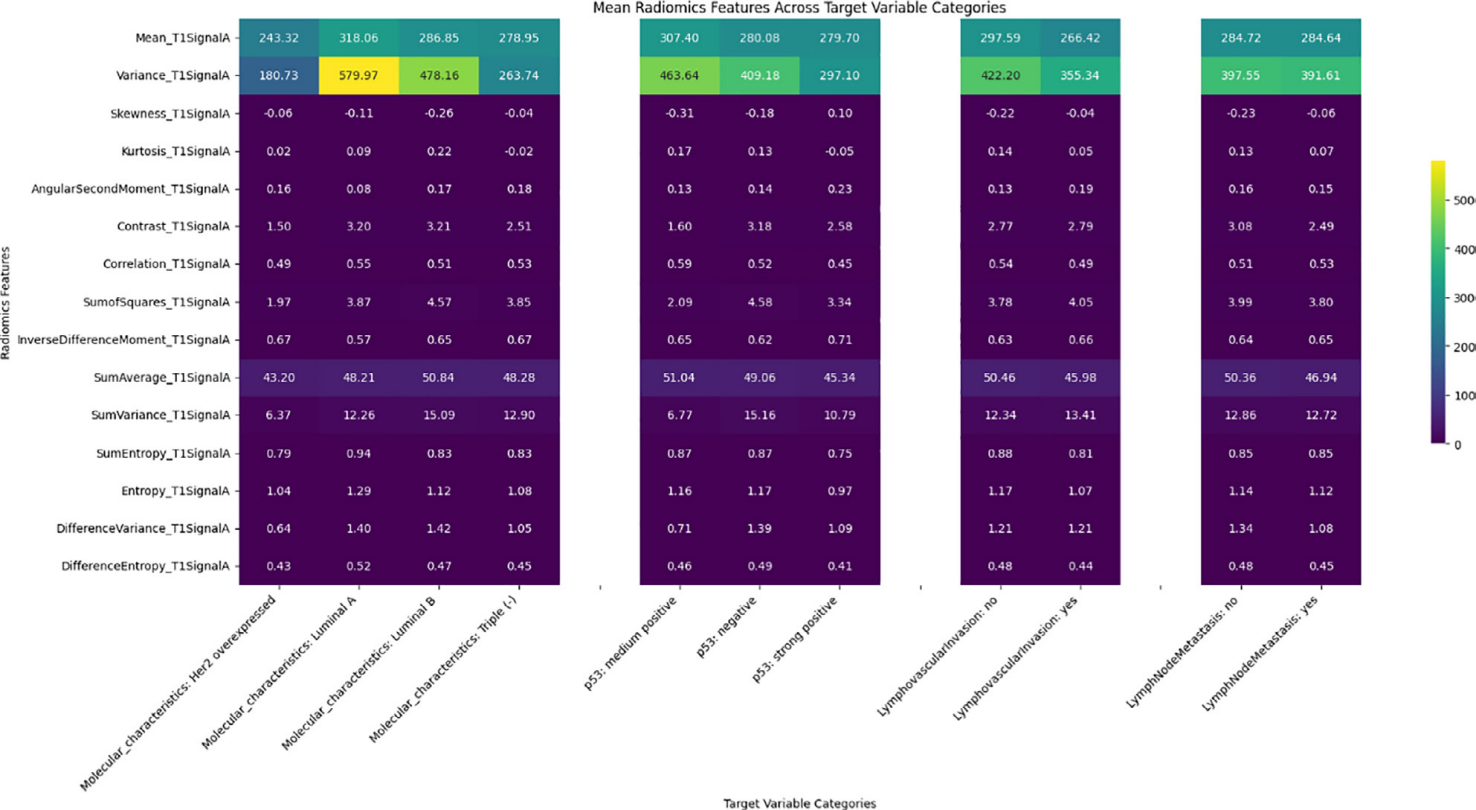
Heatmap illustrating the distribution of the T1 radiomics feature set among target variables.

**Figure 3:**
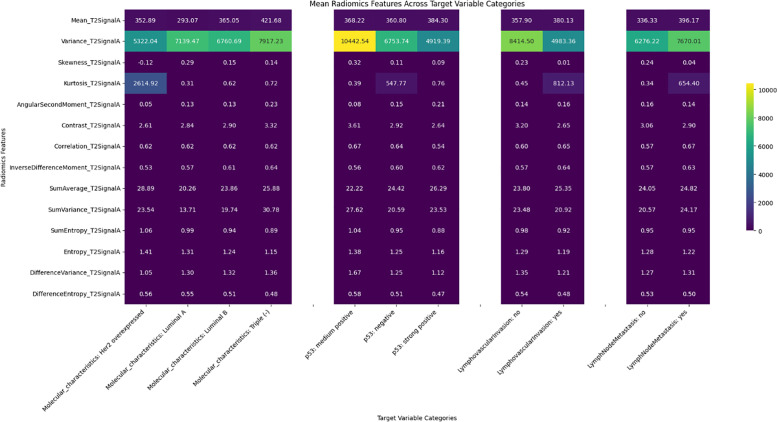
Heatmap illustrating the distribution of the T2 radiomics feature set among target variables.

**Figure 4:**
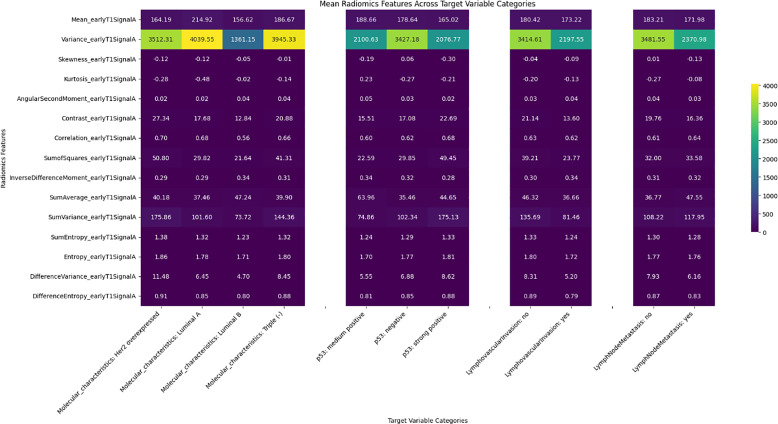
Heatmap illustrating the distribution of the early T1 radiomics feature set among target variables.

**Figure 5:**
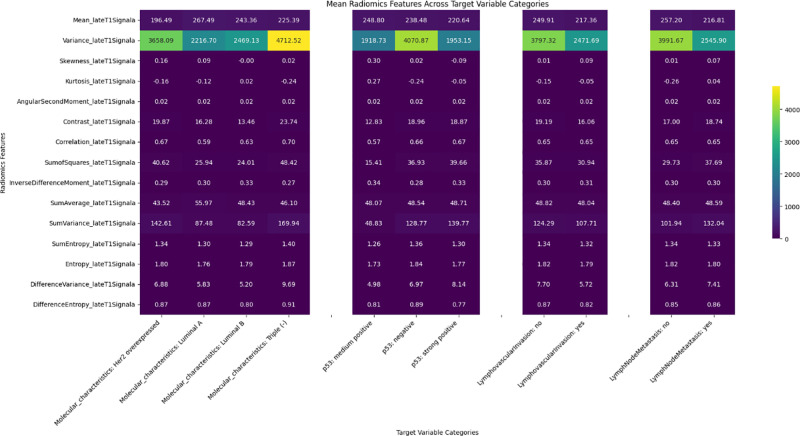
Heatmap illustrating the distribution of the late T1 radiomics feature set among target variables.

**Figure 6:**
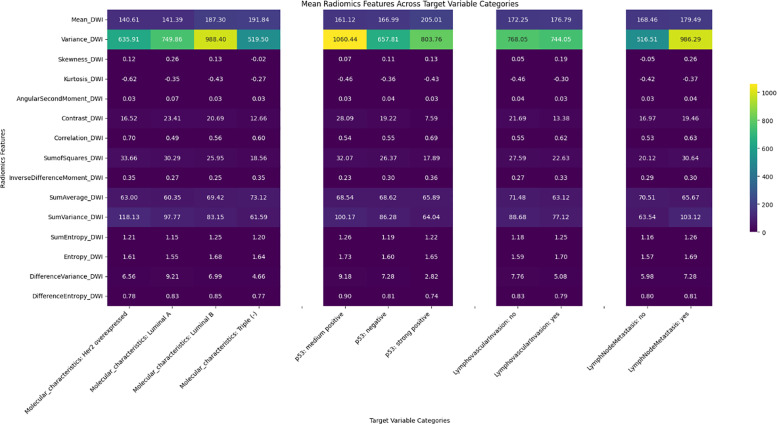
Heatmap illustrating the distribution of the DWI radiomics feature set among target variables.

Significant differences were observed in radiomics features across target variables, such as neoadjuvant response, molecular characteristics, p53 status, and lymphovascular invasion, with results shown in heatmaps (Figures 2–6). Key findings included:

- Neoadjuvant response (T1): Variance, angular second moment, contrast, sum of squares, and entropy showed significant differences between responders and non‑responders (*p*‑values: 0.0067–0.0486).
- Molecular profiling: Mean_T2SignalA distinguished Luminal A and triple‑negative cases (*p* = 0.0329). Early T1 variance separated Luminal A from Luminal B.
- p53 status: Skewness of early T1 (*p* = 0.0291) differed between negative and strong positive cases.
- Lymph node metastasis and DWI: DWI features such as mean and contrast showed significant associations, indicating a radiomic signature of treatment efficacy.

### 3.3 Prediction of molecular characteristics with radiomics feature set

Four machine learning models (Logistic Regression, Random Forest, Gradient Boosting, and XGBoost) were tested for predicting T1 molecular characteristics. XGBoost outperformed with the highest accuracy (45.71%) and AUC (67.11%), indicating balanced classification. Logistic Regression and Gradient Boosting had identical accuracies (41.43%), but Logistic Regression showed a slightly higher AUC (64.54%) and better precision. Random Forest had the highest AUC (68.01%), showing its strength in class separation.

### 3.4 Prediction of p53 status with radiomics feature set

Random Forest showed the best performance for T1 prediction with 64.29% accuracy and recall, followed by Logistic Regression (61.43%), which excelled in recall. For early T1, Gradient Boosting had the highest accuracy (64.29%) and AUC (69.67%). XGBoost showed a balanced F1 score for late T1 prediction (56.22%). Random Forest and Gradient Boosting performed well for T1 and early T1, while Logistic Regression excelled in recall, useful for minimizing false negatives in clinical settings.

### 3.5 Prediction of lymph node invasion with radiomics feature set

XGBoost was the top performer for T1 and DWI prediction, with the highest accuracy (58.93% for T1, 64.29% for DWI) and recall. Random Forest led in late T1 prediction (accuracy 67.86%, recall 67.86%). Gradient Boosting showed strong performance for early T1 prediction, with the highest accuracy (60.71%) and AUC (62.50%). Logistic Regression showed consistent recall but lower accuracy and AUC. XGBoost and Random Forest were best at balancing precision and recall.

### 3.6 Prediction of lymph node metastasis with radiomics feature set

Gradient Boosting excelled in predicting T1 and DWI metastasis, with the highest accuracy (67.86% for T1) and AUC (70.04% for T1). Logistic Regression and XGBoost had equal performance for T1, with XGBoost showing higher recall for early T1 prediction. Random Forest showed robust performance with higher recall, especially for T2 (66.60%). Gradient Boosting led in late T1 prediction (accuracy 64.29%, recall 73.91%), making it the top performer overall.

## 4. Discussion

The primary goal of this study was to examine the association between MRI texture characteristics of malignant breast masses and their histopathological and molecular features, as well as their response to neoadjuvant treatments. Our analysis revealed significant associations between MRI‑derived texture features and intratumoral heterogeneity, reflecting histopathological grading and molecular subtyping. These findings support the hypothesis that MRI texture analysis can aid in predicting pathological outcomes and treatment responses, offering potential for personalized breast cancer treatment.

Radiomics is advancing in breast imaging, distinguishing between harmful and benign lesions, identifying tumor characteristics, and predicting treatment effectiveness and recurrence [22, 23]. This study explored how MRI texture features can reveal tumor complexity, improving prediction accuracy and tailoring treatment [24]. While radiomics combined with genomics shows promise for personalized medicine, challenges remain, including the need for verified data, practical clinical use, and addressing data privacy concerns [25, 26]. Early detection is critical for improving survival rates, especially for localized breast cancer [12, 27]. Current diagnostic methods have limitations, such as limited sensitivity and invasiveness, potentially leading to missed lesion characteristics and unnecessary biopsies.

Radiomics offers a non‑invasive diagnostic approach, extracting quantitative data from imaging to reduce biopsy need and enhance personalized care. Our findings align with studies by Zhou et al. [28] and Xie et al. [29], demonstrating the ability of DCE‑MRI texture analysis to distinguish between benign and malignant lesions. This supports the growing role of radiomics in breast cancer diagnosis, as evidenced by research from Li et al. [30] and others [31], which highlights its role in improving accuracy and complementing mammography. Radiomics also adapts well across imaging modalities like tomosynthesis and ultrasound [32, 33], showing its versatility. Research by Fan et al. [34] shows that DCE‑MRI‑based radiomic features, when combined with clinical data, correlate with molecular characteristics of breast cancer.

Pre‑surgery chemotherapy is increasingly common in breast cancer management, but predicting complete response before surgery remains challenging. Our study, along with others [16, 23, 34, 35], suggests that MRI radiomic features can predict treatment response, offering a move toward non‑invasive treatment monitoring. Insights from Choudhery et al. [36] highlight DCE‑MRI radiomic features as predictors of chemotherapy response. Our research identifies a radiomic score that, combined with clinical and biological data, can predict treatment outcomes before therapy begins, potentially avoiding unnecessary treatments.

Axillary lymph node status is crucial for breast cancer prognosis, with radiomics offering predictive potential for lymph node involvement. Studies have combined radiomic and clinical‑pathological data to create predictive models [37], but the broad applicability of radiomics across different patient populations and imaging settings remains a challenge. Research by Cattell et al. [38] highlights the potential of combining deep learning and radiomics to improve model adaptability across varying imaging resolutions. This evolving synergy could strengthen predictive models.

Radiomics also offers new opportunities for predicting breast cancer recurrence risk, potentially changing treatment strategies by targeting high‑risk patients. Studies, including those by Park et al. [39] and Mazurowski et al. [40], have linked radiomic features from DCE‑MRI with recurrence risk. Our study builds on these findings, showing that combining MRI radiomic features with normal breast tissue data can enhance recurrence predictions, emphasizing the broader breast environment’s role in recurrence.

In conclusion, integrating radiomic features into clinical practice offers exciting possibilities, but challenges remain in achieving routine adoption. Consistent results across datasets, standardized feature extraction, and harmonized imaging protocols are essential for advancing the clinical utility of radiomics in breast cancer management.

Our study’s strengths include a carefully selected participant group, high‑quality MRI imaging, and collaboration with the Department of Pathology. However, limitations include a small sample size (70 cases) and the retrospective design, which may introduce bias and limit generalizability. The study’s single‑institution setting and absence of prospective validation raise questions about the applicability and real‑world effectiveness of our predictive models. Further research, particularly multi‑center studies, is needed for external validation. Despite these limitations, our findings contribute to understanding the correlation between MRI radiomic features and breast cancer characteristics, highlighting the need for larger, prospective studies to confirm their clinical utility.

## Data Availability

The datasets generated and analyzed during the current study were obtained from institutional records with approval from the local ethics committee. Due to patient confidentiality and institutional data protection policies, the data are not publicly available. Access to the datasets may be considered on a case‑by‑case basis and requires prior approval from the corresponding ethics board.
